# Genome-wide detection of selection signatures in Chinese indigenous Laiwu pigs revealed candidate genes regulating fat deposition in muscle

**DOI:** 10.1186/s12863-018-0622-y

**Published:** 2018-05-18

**Authors:** Minhui Chen, Jiying Wang, Yanping Wang, Ying Wu, Jinluan Fu, Jian-feng Liu

**Affiliations:** 1 Key Laboratory of Animal Genetics, Breeding and Reproduction, Ministry of Agriculture, College of Animal Science and Technology, China Agricultural University, Beijing, 100193 China; 2 Shandong Provincial Key Laboratory of Animal Disease Control and Breeding, Institute of Animal Science and Veterinary Medicine, Shandong Academy of Agricultural Sciences, Jinan, 250100 China

**Keywords:** Selection signatures, Chinese indigenous breed, Fat deposition, Laiwu pigs

## Abstract

**Background:**

Currently, genome-wide scans for positive selection signatures in commercial breed have been investigated. However, few studies have focused on selection footprints of indigenous breeds. Laiwu pig is an invaluable Chinese indigenous pig breed with extremely high proportion of intramuscular fat (IMF), and an excellent model to detect footprint as the result of natural and artificial selection for fat deposition in muscle.

**Result:**

In this study, based on GeneSeek Genomic profiler Porcine HD data, three complementary methods, F_ST_, iHS (integrated haplotype homozygosity score) and CLR (composite likelihood ratio), were implemented to detect selection signatures in the whole genome of Laiwu pigs. Totally, 175 candidate selected regions were obtained by at least two of the three methods, which covered 43.75 Mb genomic regions and corresponded to 1.79% of the genome sequence. Gene annotation of the selected regions revealed a list of functionally important genes for feed intake and fat deposition, reproduction, and immune response. Especially, in accordance to the phenotypic features of Laiwu pigs, among the candidate genes, we identified several genes, *NPY1R*, *NPY5R*, *PIK3R1* and *JAKMIP1*, involved in the actions of two sets of neurons, which are central regulators in maintaining the balance between food intake and energy expenditure.

**Conclusions:**

Our results identified a number of regions showing signatures of selection, as well as a list of functionally candidate genes with potential effect on phenotypic traits, especially fat deposition in muscle. Our findings provide insights into the mechanisms of artificial selection of fat deposition and further facilitate follow-up functional studies.

**Electronic supplementary material:**

The online version of this article (10.1186/s12863-018-0622-y) contains supplementary material, which is available to authorized users.

## Background

Domestic animals have been subjected to a series of strong artificial selection to meet the demand of humans, such as growth rate, body size, muscle composition and reproduction. These processes of selection have left signatures, such as reduced genetic diversity and long haplotypes, in the genomes of domestic animals. The advent of high-throughput genotyping and sequencing techniques has facilitated the detection of selection signatures in domestic animals at the genome level. Recent studies have identified candidate genes with distinct patterns of differentiation underlying the phenotypic diversity of breeds, for example, *KIT* and *MC1R* genes were related with a series of pig breed color types [1], *NR6A1*, *PLAG1* and *LCORL* genes are associated with an increased number of vertebrae and an elongation of the animal’s back [1]; *GHRL* gene was a candidate for associations with appetite and feeding behavior [2].

So far, various analytic methods have been proposed to detect different kinds of selection signatures, such as F_ST_ [3], extended haplotype homozygosity (EHH) [4], iHS (integrated haplotype homozygosity score) [5] and CLR (composite likelihood ratio) [6]. Based on these methods, genome-wide scans for signatures of diversifying selection have been successfully applied to domestic animals [7–9]. Additionally, in most cases, more than a single method is necessary in order to map the comprehensive footprint of selection in the genome. On the other hand, combining multiple methods into composite tests can lead to greater power and spatial resolution [10]. A good example is the CMS (composite of multiple signals) method [11], which combines iHS, XP-EHH (cross population extended haplotype homozygosity), F_ST_, ΔDAF and ΔiHH.

Laiwu pig is an invaluable Chinese indigenous pig breed, and distributed mainly in Laiwu district, Shandong province of China. Laiwu pig has been well-known for its high proportion of intramuscular fat (IMF) [12–14]. The unique breed feature of Laiwu pigs has come about as a result of natural and artificial selection. Therefore, it serves as an excellent model to detect footprint of selection for fat deposition in muscle, which would be beneficial for the identification of porcine genes related to IMF deposition as well as for better understanding the genetic basis of high IMF. Therefore, in this study, we investigated genomic regions under selection in Laiwu pigs using three integrative methods, F_ST_, iHS and CLR, based on genome-wide SNP genotyping data from Laiwu and Yorkshire pigs. Furthermore, in order to achieve greater power, regions identified by multiple methods were considered as candidate selected regions. Distinct patterns of selection signatures were found at loci that may contribute to domestication phenotypes, especially IMF.

## Methods

### Sample preparation and whole genome SNP genotyping

Fifty Laiwu pigs and 52 Yorkshire pigs were randomly selected from Laiwu pig conservation farm and one Yorkshire breeding farm, respectively, and genomic DNA of these samples were extracted from their ear tissues using a standard phenol/chloroform method. Both Laiwu and Yorkshire pigs belonged to one company, and used the same feeding condition except that Laiwu pigs had lower protein and higher fiber levels than Yorkshire. Genotyping was performed using GeneSeek Genomic Profiler Porcine HD BeadChip (Neogen Corporation, USA) according to the manufacturer’s instructions.

To ensure the high quality of the data, we performed the following criteria for quality control using PLINK (v1.07) [15]: (i) individuals with low genotype call rate (< 95%) were removed; SNPs were removed (ii) when the SNPs were fixed in both populations, (iii) when there were no known autosomal genomic locations in *Sus scrofa* build 10.2 [16].

Closely related individuals in the samples could bias the estimations of allele frequencies and haplotype frequencies, and thus it might mask the signature of selection. So, relatedness tests within each population were performed to ensure independence among individuals using another dataset generated by the following data filtering. Firstly, we excluded SNPs with minor allele frequency (MAF) < 0.05. Then, to reduce the dependency of SNPs in the relatedness test, we generated a pruned dataset with SNPs in approximate linkage equilibrium using the option of --indep-pairwise 50 5 0.5 in PLINK (v1.07) [15]. This option removed one of each pair of SNPs with a pairwise r^2^ > 0.5 within a window of 50 SNPs, and shifted the window by a step size of 5 SNPs. We used GCTA (V 1.26.0) [17] to compute the genetic relationship matrix [18] and removed one individual from each pair with genetic relationship higher than 0.2.

### Detection of selection signatures between Laiwu and Yorkshire

We implemented a Bayesian method [19] to estimate F_ST_ statistics between Laiwu and Yorkshire. This method assigns a weakly informative prior distribution ($Beta\left(\frac{1}{2},\frac{1}{2}\right)$) of allele frequencies. Thus, allele frequencies follow a posterior beta distribution with parameters $\alpha ={n}_A+\frac{1}{2}$ and $\beta ={n}_a+\frac{1}{2}$, here n_A_ and n_a_ indicate the counts of allele A and a in the population. The posterior distribution of allele frequencies is then used to produce the posterior distribution of F_ST_. Let ${p}_{r,l}^{(s)}$(*s* = 1, 2, ⋯, *S*) be one sample from the posterior distribution of*p*_*r*, *l*_, the frequency of allele A_*l*_ at locus *l* in population r (*r* = 1, 2, ⋯*R*). Then, a draw from the posterior distribution of F_ST_ is given by:

$$ {F_{ST}}_{\mathrm{l}}^{\left(\mathrm{s}\right)}=\frac{\sum_{\mathrm{r}=1}^{\mathrm{R}}{\left({\mathrm{p}}_{\mathrm{r},\mathrm{l}}^{\left(\mathrm{s}\right)}\right)}^2-\frac{{\left({\sum}_{\mathrm{r}=1}^{\mathrm{R}}{\mathrm{p}}_{\mathrm{r},\mathrm{l}}^{\left(\mathrm{s}\right)}\right)}^2}{\mathrm{R}}}{\left(\frac{\mathrm{R}{\sum}_{\mathrm{r}=1}^{\mathrm{R}}{\mathrm{p}}_{\mathrm{r},\mathrm{l}}^{\left(\mathrm{s}\right)}-{\left({\sum}_{\mathrm{r}=1}^{\mathrm{R}}{\mathrm{p}}_{\mathrm{r},\mathrm{l}}^{\left(\mathrm{s}\right)}\right)}^2}{\mathrm{R}}\right)} $$

From S samples of F_ST_ values, the mean of the posterior distribution of F_ST_ can be calculated and taken as the point estimate of F_ST_.

To identify highly differentiated regions, we divided the genome into 500-kb windows with a 250-kb overlap. The averaged F_ST_ value was calculated across the SNPs located within each window and was used as the test statistic. Windows falling in the top 5% of the empirical distribution were considered to be candidate regions under positive selection.

### Detection of selection signatures within Laiwu pigs

We also conducted two analytic methods, iHS and CLR tests, within Laiwu pigs. In both tests, we used the ancestral and derived alleles at each SNP locus from a previous study [20], which were determined by using four Sus species (*Sus barbatus*, *Sus celebensis*, *Sus verrucosus* and *Sus cebifrons*) and one Phacochoerus species (African warthog) as outgroups.

For iHS test, we imputed missing genotypes and inferred haplotype phase using BEAGLE (version 3.3.2) [21]. The iHS statistics were then calculated for all SNPs with MAF higher than 5%, using the software package coded by Voight and Kudaravalli [5]. This statistic compares the extent of LD between haplotypes carrying the ancestral allele and haplotypes carrying the derived allele. It integrates the EHH away from a specified core allele until EHH reaches 0.05. The integrated EHH (iHH) is denoted as iHHA or iHHD, depending on whether the core allele is ancestral or derived. The unstandardized integrated haplotype score (iHS) is defined as

$$ unstandardized\  iHS=\ln \left(\frac{iHH_A}{iHH_D}\right) $$

In the neutral model, for SNPs with comparable derived allele frequency, unstandardized his values are approximately normally distributed [5]. Thus, we split unstandardized iHS into 20 equally-sized derived allele frequency bins, i.e., with derived allele frequencies ranging from 0 to 5%, 5 to 10%, and so on. The unstandardized iHS were then normalized in each bin to obtain a zero mean and unit variance. Therefore, a large positive or negative value of iHS indicates that haplotypes carrying the ancestral or derived allele present unusually high haplotype homozygosity. To define candidate regions, we divided the genome into 500-kb windows with a 250-kb overlap and used the averaged |iHS| value in each window as the test statistic. Windows at the top 5% of the empirical distribution were considered to be candidate regions of positive selection.

A recent selective sweep causes a skew of frequency spectrum at linked sites, such as reduced genetic diversity and an excess of derived alleles at high frequencies [22, 23]. CLR test compares a neutral model for allele frequency spectrum with a selective sweep model. In the neutral model, the probability of allele frequency spectrum is derived from the background pattern of variation in the genome. We used SweepFinder2 [24] to calculate the CLR statistics for sites every 20 kb across the genome. To define candidate region, we divided the genome into 500-kb windows with a 250-kb overlap. In each window, the maximum CLR was used as the test statistic, following a previous approach [25]. Windows at the top 5% of the empirical distribution were considered to be candidate regions of positive selection.

To further control the false positive rates of the detection of selection signatures, genomic regions identified by at least two methods were used in further analyses.

### Functional characterization of genomic regions under selection

Gene contents in candidate selected regions were retrieved from Ensembl Genes 89 Database using BioMart (http://asia.ensembl.org/biomart/martview/). QTLs (quantitative trait loci) were downloaded from the Pig QTLdb (http://www.animalgenome.org/cgi-bin/QTLdb/SS/download?file=bedSS_10.2), and compared with those selected regions based on the putative location of the QTLs. Furthermore, bioinformatics analyses including Gene Ontology (GO) and KEGG pathway enrichment analyses were performed using DAVID 6.8 (https://david.ncifcrf.gov/#, Oct. 2016) to reveal the potential biological function of candidate genes harbored in selected regions. The enriched GO terms and pathways with *P*-values < 0.05 were used for further analysis in our study.

## Results

### Information of chip data

A total of 68,516 SNPs were genotyped by GeneSeek Genomic Profiler Porcine HD BeadChip. After a series of quality control, 50,432 autosomal SNPs were remained for the 50 Laiwu pigs and 52 Yorkshire pigs. The average distance between adjacent SNPs was 51.4 kb, with the standard deviation of 79.7 kb. Therefore, we chose a window size of 500 kb to identify candidate region so that on average there were about 10 SNPs in per window.

In the process of relatedness test to ensure no common ancestry for 3 generations among sample used in selection signatures detection, 35 Laiwu pigs and 26 Yorkshire pigs were excluded because of their close relationships (genetic relationship higher than 0.2). The genetic relationships between individuals before and after removing these closely individuals are shown in Additional file 1: Figure S1. Finally, 15 Laiwu pigs and 26 Yorkshire pigs were left for the detection of selection signatures. These samples sizes were sufficient to estimate allele frequencies, based on which F_ST_ and CLR were calculated. Taking account for the small effective population sizes in pig breeds, the sample sizes should also be sufficient for iHS in comparison with human populations, where 40 chromosomes could provide moderate power in iHS test in Yoruba in Ibadan, Nigeria [25].

### Genome-wide scanning for selection signatures

**Fig. 1:**
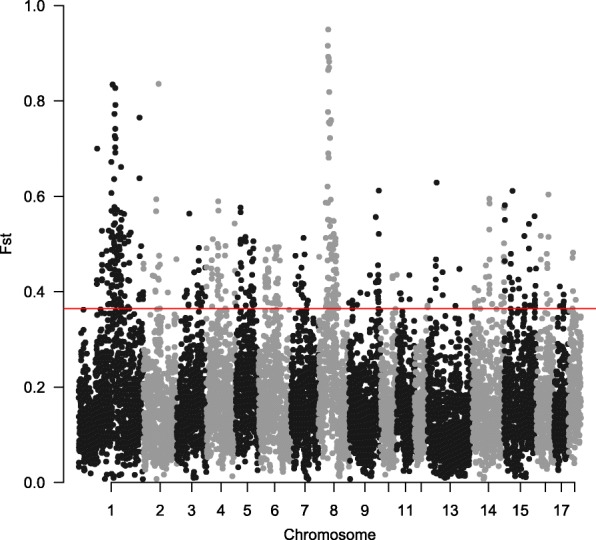
Genome-wide distribution of selection signatures detected by F_ST_ on 18 chromosomes. Red line displays the threshold levels of 5%

To detect signatures of positive selection in Laiwu pigs, we used three statistical analysis methods. We first used F_ST_ statistic to make comparisons between Laiwu pigs and Yorkshire. F_ST_ values for SNPs ranged from 0.006 to 0.952, with an average of 0.169 and standard deviation of 0.167. The genome-wide distribution of the averaged F_ST_ for 500-kb window is shown in Fig. 1. A total of 486 candidate windows (Additional file 2: Table S1), falling in the top 5% of the empirical distribution, were considered to be candidate regions under positive selection. Additionally, among these candidate windows, there were two clusters with extremely high differentiation. The first cluster was on *Sus scrofa* chromosome 1 (SSC1), with the window 165.00–166.00 Mb presenting F_ST_ value of 0.83. The second cluster was on SSC8, with the window 46.50–47.00 Mb presenting the highest F_ST_ value of 0.95.

**Fig. 2:**
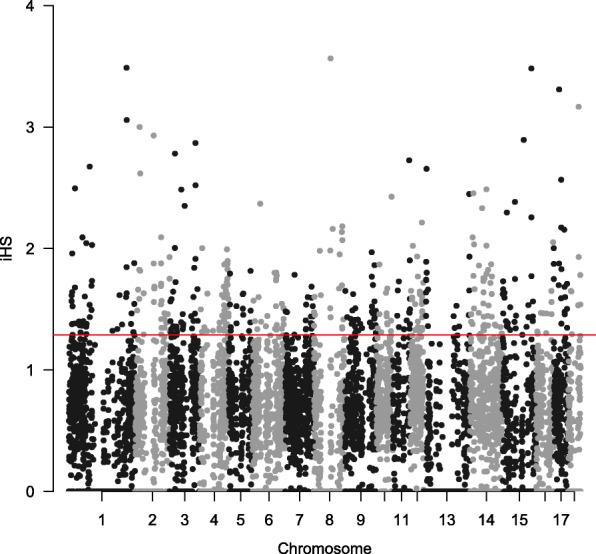
Genome-wide distribution of selection signatures detected by iHS on 18 chromosomes. Red line displays the threshold levels of 5%

**Fig. 3:**
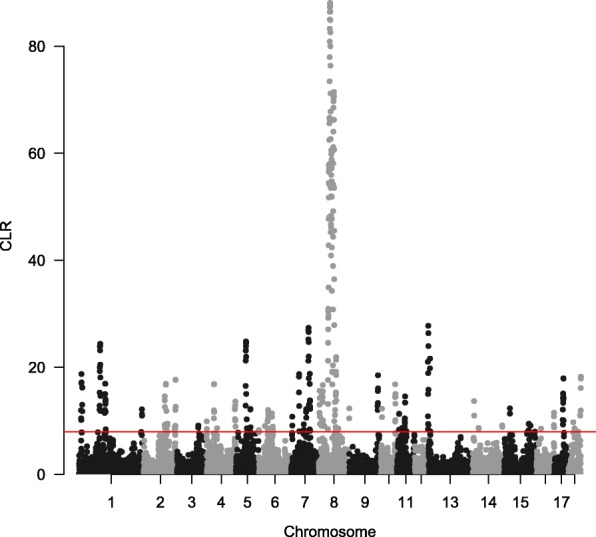
Genome-wide distribution of selection signatures detected by CLR on 18 chromosomes. Red line displays the threshold levels of 5%

We also ran two within-population methods, iHS and CLR, to detect selection signatures within Laiwu pig population. Figure 2 shows the genome-wide distribution of iHS values. The genome-wide mean |iHS| value was 0.43, and the highest average |iHS| value was 3.48 for the window on SSC15 (spanning 134.25–134.75 Mb). There were 480 windows (Additional file 3: Table S2) at the top 5% of the empirical distribution that were considered to be candidate regions of positive selection. Figure 3 illustrates the CLR statistic against the genomic position for Laiwu pigs. The result provided strong evidence of a selective sweep on SSC8, with a cluster of extreme signals and two windows (54.50–55.00 Mb and 54.75–55.25 Mb) harboring the largest CLR value (88.09). Across the genome, we observed 490 windows (Additional file 4: Table S3) with extreme CLR values that were considered candidate regions of positive selection.

### Overall comparison of selection signatures between methods

**Fig. 4:**
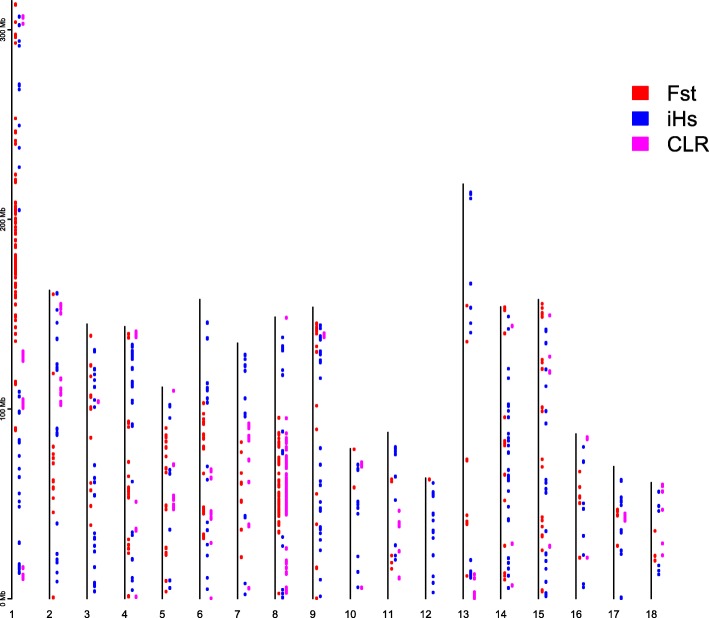
Genomic distribution of selection signatures detected by F_ST_, iHS and CLR on 18 chromosomes

The distribution of overall candidate selected regions along the genome has been shown in Fig. 4. Genomic regions identified by at least one method covered 465 Mb. Among them, the candidate regions obtained by both F_ST_ and CLR covered 29.75 Mb; the candidate regions obtained by both F_ST_ and iHS covered 9 Mb; the candidate regions obtained by both CLR and iHS covered 5.5 Mb. Besides, there was one region 2.75–3.0 Mb on SSC8 identified by all three methods.

In order to decrease the false positive regions identified, candidate regions obtained by at least two of the three methods were considered to be final candidate regions of positive selection. Totally, there were 175 candidate regions (Additional file 5: Table S4) obtained by at least two of the three methods, covering 43.75 Mb genomic regions and corresponding to 1.79% of the autosome sequence. The numbers of candidate selected regions are uneven distributed across the genome. The biggest number of regions were observed on SSC8 (86 regions), while no ones were found on SSC12.

### Functional characterization of candidate regions

Totally, 438 genes within the identified selected regions were retrieved from Ensembl Genes 89 Database, including 395 protein-coding genes, seven miRNA, three pseudogenes, nine snoRNA, 16 snRNA, three rRNA, and four miscRNA (Additional file 6: Table S5). We noted that some selected regions were mapped in the gene desert, which accounted for approximately 25.14% of all selected regions, indicating the important function of non-coding sequence in the selection process. Though some genes are yet not annotated, gene annotation of selected regions revealed a list of functionally important genes, such as *NPY1R*, *NPY5R*, *PIK3R1* and *JAKMIP1* for feed intake and fat deposition, *ESR1* and *PTHLH* for reproduction, and *CXCL2*, *CXCL8* and *TLR2* for immune response. Table 1 summarizes ten candidate selected regions harboring interesting genes.

**Table 1:** Summary of the 10 selected regions harboring interesting candidate genes

SSC	Position (Mb)	Methods	Candidate genes	Function
1	16.50–16.75	iHS, CLR	*ESR1*	Litter size
5	49.00–49.25	F_ST_, CLR	*PTHLH*	Mammary gland and nipple development
6	45.00–45.25	F_ST_, CLR	*TGFB1*	Fat deposition
6	45.50–45.75	F_ST_, CLR	*GSK3A*	glycogen synthase
8	45.25–45.5	F_ST_, CLR	*CPE*	Fat deposition
8	2.75–3.00	F_ST_, iHS, CLR	*JAKMIP1*	Feed intake
8	55.25–55.50	F_ST_, CLR	*NPY1R*, *NPY5R*	Stimulation of appetite
8	74.00–74.25	F_ST_, CLR	*CXCL2*, *CXCL8*	Immune response
8	79.75–80.00	F_ST_, CLR	*TLR2*	Immune response
16	50.25–50.50	F_ST_, iHS	*PIK3R1*	Energy balance

We further investigated the functions associated with the annotated genes undergoing positive selection by analyzing over-represented GO terms and pathways using DAVID. The significant enriched GO terms and KEGG pathways are shown in Additional file 7: Table S6. A total of 34 significant enriched GO terms with *P-*value < 0.05 were observed, including 16 terms for functional terms (BP) category, 12 terms for molecular function (MF) category, and five terms for cellular component (CC) category. For BP category, most of the terms were involved in regulation of basic metabolic process, cell proliferation, signaling pathway, such as regulation of cell proliferation (0042127), epidermal growth factor receptor signaling pathway (GO:0007173), neuropeptide signaling pathway (0007218). Besides, some of these significant enriched terms were involved in feed behavior and immune response. The 10 most important enriched functional BP terms are summarized in Table 2.

**Table 2:** Summary of 10 enriched functional biological process (BP) terms relevant to phenotypic traits

Term	Count	*P*-value	Fold enrichment
GO:0070098~chemokine-mediated signaling pathway	6	0.0002	10.59
GO:0007173~epidermal growth factor receptor signaling pathway	4	0.0077	9.68
GO:0042127~regulation of cell proliferation	7	0.0096	3.84
GO:0007218~neuropeptide signaling pathway	5	0.0123	5.54
GO:0002690~positive regulation of leukocyte chemotaxis	3	0.0161	15.08
GO:0050714~positive regulation of protein secretion	3	0.0212	13.07
GO:0032496~response to lipopolysaccharide	5	0.0250	4.47
GO:0033138~positive regulation of peptidyl-serine phosphorylation	4	0.0258	6.22
GO:0097466~glycoprotein ERAD pathway	2	0.0450	43.56
GO:0007631~feeding behavior	3	0.0473	8.52

In addition, there were 18 significant pathways enriched (Additional file 8: Table S7), such as steroid hormone biosynthesis (ssc00140), metabolic pathways (ssc01100), chemokine signaling pathway (ssc04062) and salivary secretion (ssc04970).

### Identifying QTL overlapping with candidate selected regions

We downloaded 16,516 QTLs from the pig QTL database (Release 31, Dec 30, 2016) and identified any overlapping of the candidate selected regions with those QTLs. Consequently, 1519 porcine QTLs (Additional file 9: Table S8) were detected to be overlapped with the candidate selected regions identified. Interestingly, we found that the number of QTLs relating to meat and carcass traits was especially greater than others, with a proportion of 52.92%, indicating that selection for meat quality during Laiwu pigs breeding has left a detectable footprint in the pig genome.

## Discussion

In this study, we implemented tests to detect the genome-wide footprints left by natural and artificial selection in Laiwu pigs. Our results revealed a number of regions showing signatures of positive selection. Functional analyses on candidate selected regions supported that these regions had been under selection for fat deposition and the regulation of energy balance. Furthermore, we also found evidence for selection on other traits, such as reproduction and immune response.

With the molecular tools developed for pigs as well as other livestock species, a variety of methods have been developed for detecting different kinds of selection signatures. According to the information used in the test, these methods can be grouped into three categories: population differentiation, site-frequency spectrum and linkage disequilibrium [26, 27]. All the methods detect candidates of different types of selection, and are essential to construct a comprehensive selection map for the pig genome. Therefore, in the present study, we implemented three complementary methods (F_ST_, iHS and CLR) to comprehensively identify candidate regions of positive selection. As shown in Figs. 1, 2 and 3, different selected regions were obtained by these methods. Comparatively speaking, F_ST_ and CLR have the highest overlap rate, with overlapped selected regions of 29.75 Mb, whereas F_ST_ and iHS, and CLR and iHS only have overlapped selected regions of 9 Mb and 5.5 Mb, respectively. However, there was only 0.25 Mb (2.75–3.0 Mb on SSC8) identified by all the three methods. It is possible that different methods emphasize different information in the data and are sensitive to different categories of selection signatures. Specifically, F_ST_ is more powerful for detecting complex events, such as selection on standing variation [28]; iHS test has advantages in exploring selective sweeps with variants at moderate frequencies [29]; and, CLR test is more sensitive to selective sweeps with variants approaching fixation in the population [30]. It is worthy to note that the ascertainment bias in genotyping data might distort the genome-wide distribution of allele frequencies, as well as the estimations of F_ST_ and CLR. The effect of ascertainment bias can be mitigated by correcting the allelic distribution using statistical methods [6, 31, 32]. However, these methods are not suitable for our study. The GeneSeek Porcine HD BeadChip used in this study was designed mainly using SNPs identified in wild boars and European breeds (https://support.illumina.com.cn/downloads/geneseek-ggp-porcine-hd-product-files.html). Since Chinese pigs and European pigs split over 1.2 million years ago [33], these two populations are highly differentiated, as also shown in previous studies [16, 34]. Therefore, the ascertainment scheme in the design of the SNP chip cannot help correct the ascertainment bias effect on Laiwu pigs, a Chinese indigenous breed.

In comparison with previous genome-wide scans for selection signatures in Chinese indigenous pigs, we find a number of overlapping signatures between Laiwu and other Chinese breeds. Take the cluster of signals detected by both F_ST_ and CLR on SSC8 for example, previous analysis [35] in Tongcheng pigs, another typical Chinese indigenous breed, found a series of windows in this region with very low genetic diversity and being highly differentiated from Chinese wild boars, indicative of a selective sweep. Another study on Chinese Rongchang pigs also identified a cluster of selection signatures in this region [36]. Except for this region, we also find clusters of overlapping selection signatures between Laiwu and Tongcheng or Rongchang on other chromosomes, such as the cluster of F_ST_ signals on 148.25–149.75 Mb of SSC1.

The primary goal of this study is to identify putative candidate genes involved in Laiwu pigs, which is an invaluable Chinese typical indigenous pig breed with extremely high proportion of IMF. In accordance with the characteristics of Laiwu pigs, a series of genes relevant to phenotypic traits, especially ones regulating feed intake and fat deposition, were annotated in the candidate regions of positive selection, such as *NPY1R*, *NPY5R*, *PIK3R1* and *JAKMIP1*. Additionally, some of these functional genes were also detected as being under selection or related with energy balance by previous studies.

The arcuate nucleus of hypothalamus has two sets of neurons, which are two central regulators in maintaining the balance between food intake and energy expenditure [37]. One set of neurons produce agouti-related protein (AGRP) and neuropeptide Y (NPY); another set of neurons produce pro-opiomelanocortin (POMC) and cocaine- and amphetamine-related transcript (CART). The NPY and AGRP are orexigenic, promoting food intake and reducing energy expenditure, while the POMC and CART produce the opposite anorexigenic effect [38]. In this study, we identified a list of genes involved in the actions of these two sets of neurons.

In the cluster of signals identified by both FST and CLR, we identified the *NPY1R* and *NPY5R* genes on 55.25–55.50 Mb of SSC8, which were two receptors of NPY. Previous study showed that the *NPY1R* and *NPY5R* appeared to be candidates for mediating the orexigenic effects of NPY [39]. *NPY1R* and *NPY5R* double knockout mice behaved as hypophagic, although their body weight increased due to decreased energy expenditure [40]. Genetic association analyses also revealed that these two genes were related to food intake [41] and obesity [42, 43] in humans. The signals of selection on the *NPY1R* and *NPY5R* genes have also been found in multiple Chinese pig breeds, such as Tongcheng [35], Rongchang and Jinhua [44].

Peripheral endocrine signals, including leptin and insulin, can regulate the energy balance by modulating the activity of arcuate POMC/CART and NPY/AGRP neurons [37]. One part of the effect of leptin and insulin on arcuate neurons was via the activation of phosphoinositide-3-kinase (PI3K) signaling [45–47]. FST and iHS identified signals of selection on the *PIK3R1* gene, which encoded three regulatory units of PI3K, including p85α, p50α and p55α. Previous studies [47, 48] showed that they played important roles in leptin- and insulin-induced regulation of energy homeostasis.

The NPY/AGRP neurons also have an inhibitory effect on the POMC/CART neurons through the release of GABA [37]. *JAKMIP1* is a RNA-binding protein associated with GABAB receptors [49], one of two classes of GABA receptors. It can regulate the cellular levels of GABAB R2 subunits, and may have effects on the production of GABAB receptors [49]. Therefore, the *JAKMIP1* gene has a potential effect on the regulation of energy balance. Previous genome-wide association study on residual feed intake in quality chickens found that the polymorphism in the intron of *JAKMIP1* gene explained 9.71% of phenotypic variance [50].

High litter size and good maternity performance were other specific features of Laiwu pigs. Gene annotation on candidate regions also resulted in some genes associated with reproduction traits. For instance, both CLR and iHS identified selection signals of *ESR1* gene on SSC1. Estrogen is known for its role in pregnancy. The primary mechanism of its action is mediated through its receptors, i.e., *ESR1* and *ESR2*. Association studies [51–53] have proven the effect of *ESR1* on litter size in pigs. Besides, FST and CLR identified the *PTHLH* gene in the cluster of signals on SSC5. This gene is essential for mammary gland development during embryogenesis [54] and nipple development during pregnancy and lactation [55]. Signals of selection on this gene have also been identified in European pig breeds [2] and Chinese Rongchang pigs [36]. A previous study [56] found that this gene was significantly related to teat number and inverted teat phenotype.

## Conclusion

In summary, we report here the identification of selection signatures in the genome of Laiwu pigs, a typical Chinese indigenous breed. The evidence presented here demonstrates that Laiwu pigs have been under strong selection on fat deposition. Besides, genomic regions under selection also contribute to reproduction and health traits. These results provide insight into the genome evolution and selection mechanisms in Chinese indigenous pig breeds.

## Additional files

Additional file 1: **Figure S1.** Genetic relationships between individuals before and after removing closely related individuals. (A) genetic relationship in Laiwu pigs before removing closely related individuals; (B) genetic relationship in Laiwu pigs after removing closely related individuals; (C) genetic relationship in Yorkshire before removing closely related individuals; (D) genetic relationship in Yorkshire after removing closely related individuals. (PDF 15 kb)

Additional file 2: **Table S1.** Summary of selected regions under positive selection detected by F_ST. (XLSX 51 kb)_

Additional file 3: **Table S2.** Summary of selected regions under positive selection detected by iHS (XLSX 47 kb)

Additional file 4: **Table S3.** Summary of selected regions under positive selection detected by CLR. (XLSX 23 kb)

Additional file 5: **Table S4.** Summary of overlapping candidate regions under positive selection detected by at least two of the three methods F_ST_, iHS and CLR. (XLSX 16 kb)

Additional file 6: **Table S5.** Gene contents in candidate regions under positive selection. (XLSX 38 kb)

Additional file 7: **Table S6.** Significantly enriched Gene Ontology terms of candidate genes. (XLSX 12 kb)

Additional file 8: **Table S7.** Significantly enriched pathway of candidate genes. (XLSX 10 kb)

Additional file 9: **Table S8.** Summary of QTL overlapped with candidate selected regions. (XLSX 150 kb)

Additional file 10: The ped PLINK file of SNP genotyping data for the 50 Laiwu pigs and 52 Yorkshire pigs. (TXT 21549 kb)

Additional file 11: The map PLINK file of SNP genotyping data for the 50 Laiwu pigs and 52 Yorkshire pigs. (TXT 1544 kb)

## Abbreviations

CLR: Composite likelihood ratio
EHH: Extended haplotype homozygosity
GO: Gene ontology
iHS: Integrated haplotype homozygosity score
IMF: Intramuscular fat
LD: Linkage disequilibrium
MAF: Minor allele frequency
SSC: *Sus scrofa* chromosome
