# Supplementary figures and images for: Genome-wide detection of selection signatures in Chinese indigenous Laiwu pigs revealed candidate genes regulating fat deposition in muscle
Source: BMC Genet. 2018 May 18;19:31. doi: 10.1186/s12863-018-0622-y (PMC5960162; doi:10.1186/s12863-018-0622-y)

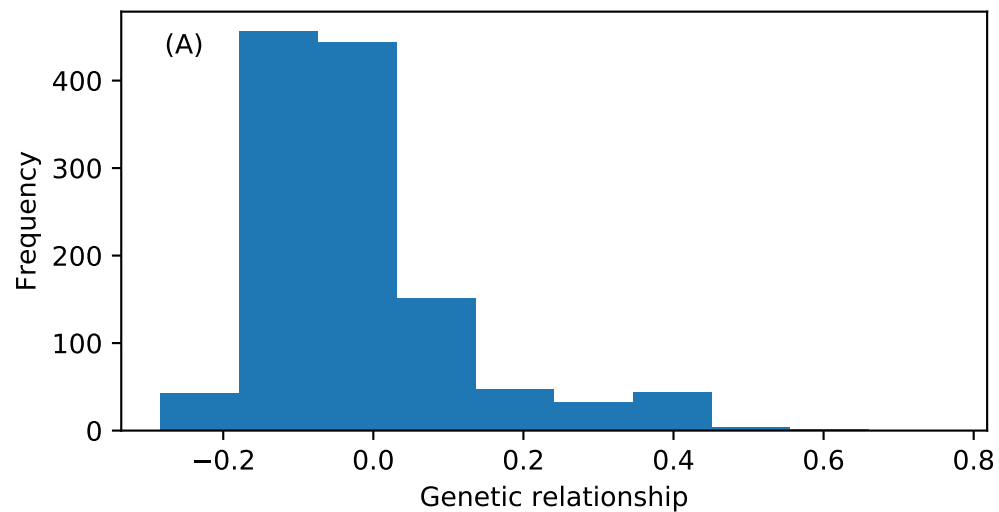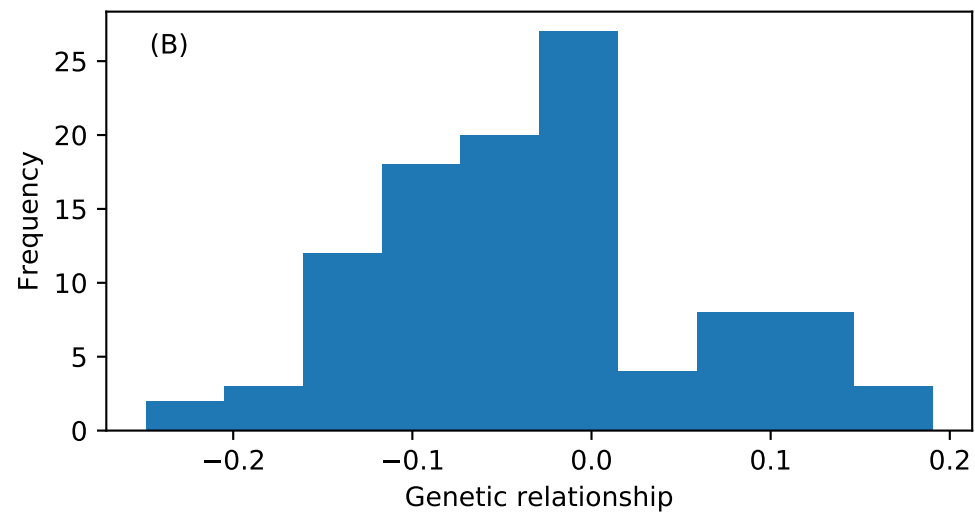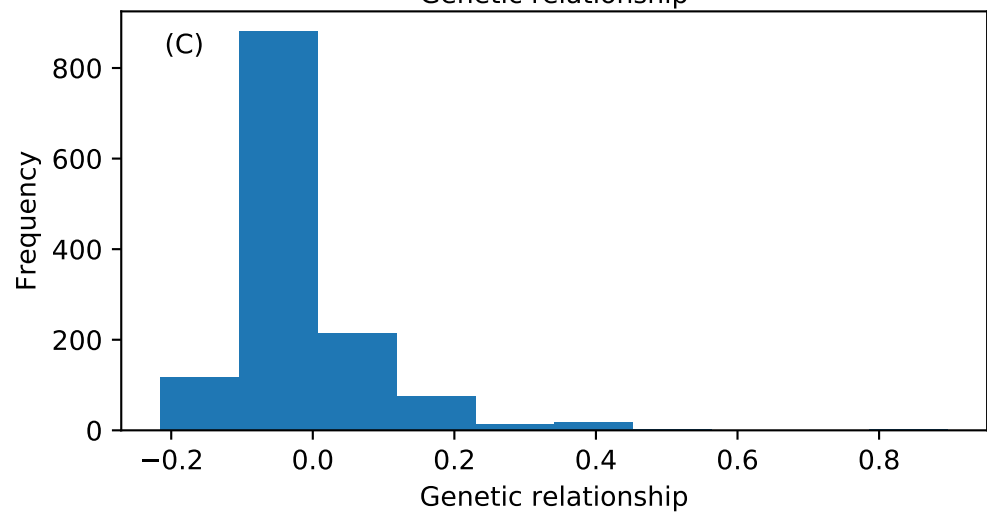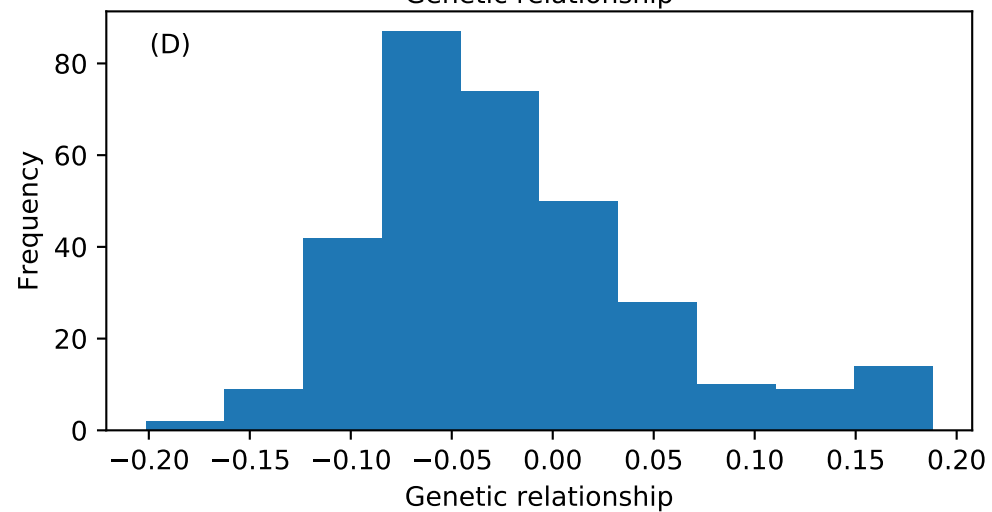

Supplement: Supplementary file 1 — Figure S1. Genetic relationships between individuals before and after removing closely related individuals. (A) genetic relationship in Laiwu pigs before removing closely related individuals; (B) genetic relationship in Laiwu pigs after removing closely related individuals; (C) genetic relationship in Yorkshire before removing closely related individuals; (D) genetic relationship in Yorkshire after removing closely related individuals. (PDF 15 kb) [file 12863_2018_622_MOESM1_ESM.pdf]
